# A tryst of ‘blood pressure control- sex- comorbidities’: the odyssey of basic public health services in Yunnan in quest for truth

**DOI:** 10.1186/s12889-023-17157-7

**Published:** 2024-02-16

**Authors:** Linhong Pang, Lakshme Kottu, Zihong Guo, Min Ma, Huadan Wang, Yajing Zhao, Mingjing Tang, Yi Shi, Wei Liu, Xia Wu, Junjie Song, Manli Sun, Daphne Merkus, Md Misbahul Ferdous, Liping He, Lin Duo

**Affiliations:** 1 Fuwai Yunnan Cardiovascular Hospital, Affiliated Cardiovascular Hospital of Kunming Medical University, 650221 Kunming, China; 2 School of Public Health, Kunming Medical University, 650500 Kunming, China; 3 Division of Experimental Cardiology, Erasmus university medical center, 3015GD Rotterdam, The Netherlands; 4 Walter Brendel Center of Experimental Medicine (WBex), LMU, 81377 Munich, Germany; 5 The Third People’s Hospital of Longgang District, 518083 Shenzhen, China; 6 Lepu Medical Technology (Beijing) Co., Ltd, 102200 Beijing, China

**Keywords:** Hypertension, Comorbidities, Sex, Blood pressure control, BPHS

## Abstract

**Background:**

The Basic Public Health Service (BPHS), a recently announced free healthcare program, aims to combat the most prevalent Noncommunicable Disease-“Hypertension” (HTN)-and its risk factors on a nationwide scale. In China, there is a rife that HTN less impacts women during their lifetime. We, therefore, aimed to evaluate the sex disparity in hypertension patients with comorbidities among south-west Chinese and the contribution of BPHS to address that concern.

**Methods:**

We have opted for a multistage stratified random sampling method to enroll hypertensive patients of 35 years and older, divided them into BPHS and non-BPHS groups. We assessed the sex disparity in HTN patients with four major comorbidities- Dyslipidemia, Diabetes Mellitus (DM), Cardiovascular Disease (CVD), and Chronic Kidney Disease (CKD), and descriptive data were compiled. Odds ratios from logistic regression models estimated the effectiveness of BPHS in the management of HTN with comorbidities.

**Results:**

Among 1521 hypertensive patients,1011(66.5%) were managed in the BPHS group. The proportion of patients who had at least one comorbidity was 70.7% (95% confidence interval [CI]: 66.3-76.8%), patients aged 65 years and older were more likely to have coexisting comorbidities. Participants who received the BPHS showed significant blood pressure (BP) control with two comorbidities (odds ratio [OR] = 2.414, 95% CI: 1.276–4.570), three or more (OR = 5.500, 95%CI: 1.174–25.756). Patients with dyslipidemia and DM also benefited from BPHS in controlling BP (OR = 2.169, 95% CI: 1.430–3.289) and (OR = 2.785, 95%CI: 1.242–6.246), respectively. In certain high-income urban survey centers, there was sex differences in the HTN management provided by BPHS, with men having better BP control rates than women.

**Conclusions:**

Perhaps this is the first study in China to succinctly show the effectiveness and sex disparity regarding “management of hypertensive comorbidities”. This supports that the BPHS program plays a pivotal role in controlling BP, therefore should recommend the national healthcare system to give women a foremost priority in BPHS, especially to those from low-socioeconomic and low-scientific literacy regions.

**Supplementary Information:**

The online version contains supplementary material available at 10.1186/s12889-023-17157-7.

## Introduction

Hypertension (HTN) is a major modifiable risk factor and a leading cause of mortality and premature deaths among the NCD’S [1–2]. It is reported that more than 1 billion people are affected globally [3]. Managing HTN is a significant public health concern [4]. The prevalence of HTN is increasing in low and middle-income countries (LMICs) than in high-income countries [3–5].

China has approximately 245 million HTN patients, with treatment and control rates of only 40.7% and 15.3%, respectively [6]. HTN is not only a risk factor for many unfavorable clinical outcomes but also has an adverse effect on other conditions like diabetes mellitus (DM), chronic kidney disease (CKD), cardiovascular diseases (CVD), stroke, and loss of cognitive function [7–10]. Therefore, even modest improvements in the management of raised BP might have a significant long-term positive impact, especially in patients with underlying comorbid conditions [7]. It is reported that two-thirds of hypertensive patients had underlying comorbidities such as DM, CKD, CVD, and dyslipidemia [11].

BP is not only diurnal [12–13] but also a dimorphic variable that can change at different rates in males and females throughout their life span [13–14]. These variations are caused by a combination of genetically (sex-related) and psychosocially (gender-related) determined variables [15–16], but overall, men are more likely to have other coexisting conditions in China [17]. The prevalence of HTN differs between males and females with comorbidities, as does their awareness of the condition [6, 18–21]. Sex disparities in the treatment of HTN with comorbidities, however, have not received enough consideration [22–23]. Sex disparities in HTN care, which have been overlooked, would hinder the Sustainable Development Goal (SDGs) of reducing non-communicable diseases (NCDs) mortality by one-third [24–25]. It would be challenging to reach SDG’s goal of 3.0 in Yunnan Province (a low-income region in south-west China) [26].

Based on the American College of Cardiology (ACC)/American Heart Association (AHA) hypertension guidelines [27] and the Chinese guidelines [28–29], it was prioritized to take into account HTN along with other comorbidities like CVD, heart failure, stroke, CKD, DM, and dyslipidemia when considering secondary prevention. The Basic Public Health Service (BPHS) program is a nationwide free primary health care policy initiated by the Chinese government, and one of its main pillar is the management of HTN [30–31]. In the past, numerous publications from China have focused on different risk factors that can be modified to reduce hypertension [32–35]. These were followed by reports on how the BPHS policy launch attempted to address and combat these risk factors through proper lifestyle and treatment adherence guidance [33, 36–37]. However, a meta-analysis revealed that the efficacy of managing HTN in individuals with comorbidities through BPHS program still remains a question [38]. In addition, there are currently no data outlining how health services can control the prevalence of HTN with comorbidities in the south-west Chinese population across sex.

In our study, which was conducted in Yunnan province, the main objectives were to examine how sex differences play a role in the management of HTN, especially in hypertensive populations with underlying comorbidities, as well as how the new health policy ‘BPHS’ introduced by the government is capable of managing this issue.

## Methods

### Study design, setting, and participants

This cross-sectional study was carried out among HTN patients between January 2021 to December 2021 in Yunnan, a province with a population of 47.2 million. Yunnan was chosen as the survey location for the national monitoring project because it is unique in several ways. Its Gross Domestic Product (GDP) per capita is 5068 USD, which is markedly less than the average GDP of the rest of China, which is 8777 USD. It is a home for ethnic minorities (25 out of 55 official ethnic minorities). Furthermore, this province has a diverse terrain that includes mountain ranges (94% of the landscape), turbulent rivers, idyllic lakes, and terraced hills. Yunnan Center has adhered to the guidelines concerning its study design of sites, sampling scheme, and sampling size mentioned by the “Chinese Cardiovascular Disease and Related Risk Factors Monitoring Project“(Chinese equivalent to the Demographic and Health Survey, DHS), organized by the National Center for Cardiovascular Disease (NCCD). As we needed a sample size representative of the entire Yunnan province; we employed a multistage stratified random sampling method representing the entire adult population in both sex [33]. A total of 9600 residents aged 18 years and older were randomly selected from 4 urban areas and 4 rural counties in Yunnan Province for cardiovascular disease screening (for more details, see Fig. 1 in Supplementary file 1).

In this survey, 2764 cases of HTN among 9600 adults met the diagnostic criteria outlined in the International Society of Hypertension (ISH) [7]. According to the recommendations of the BPHS health care policy, the target group for HTN management is individuals who are 35 years of age and older with past history of hypertension or who were already on anti-hypertensive medications [30–31]. Therefore, newly diagnosed patients who are younger than 35 were excluded for comparability purposes. Subsequently 1521 participants, both men and women aged 35 years old or above with or without comorbidities were included in this study.

We linked these 1521 patients’ unique identifiers to the BPHS electronic system in Yunnan Province, 1011 patients who received the services of BPHS management were included in the ‘BPHS group’, and those who did not receive were included in the ‘non-BPHS group’ (see Fig. 2 in Supplementary file 1). This study has been approved by the Ethics Review Boards of NCCD and informed consent and electronic signatures were obtained on-site for all participants. An overview of the study was presented in Supplementary file 2.

### Data collection and variables

#### Data collection

We created a uniform data management platform and used iPads with a unified preconfigured APP to save information from surveys on-site, which is in accordance with the NCCD manual. The face-to-face interviews using a standard structured questionnaire designed by NCCD were collected by a group of well-trained investigators (physicians, nurses, medical college students, and pharmacists) at each study center.

### Independent variables

Most of the independent variables used in this study were categorical, representing demographic characteristics, lifestyle, history of hypertension, and history of comorbidities, amongst whom the ethnic groups are divided as the Han (nationwide ethnic majority) and ethnic minorities. Ages were categorized as “35–44, 45–54, 55–64, and ≥ 65 years”. Information regarding sex, marital status, residence, and employment status were also collected. Annual household income was classified as low income (lower than the moderate level of < 4487 USD/year) and high income (4487 USD/year and above). The educational level was divided into primary or lower school, middle school, or higher. Current smokers are defined as those who have smoked one cigarette per day for more than six months [39], and current drinkers are those who drink alcohol at least once a week [40]. Further, the investigators asked whether patients had received the following lifestyle modification health education in the past two weeks (including reduced sodium intake, increased physical activity, weight loss, tobacco cessation, reduced alcohol intake, and stress reduction intervention), which were consistent with ISH and ACC guidelines [7, 27]. The study included dichotomous variables for history of HTN, anti-hypertensive medications, and history of comorbidities. Anti-hypertensive drugs are divided into five major categories: angiotensin-converting, enzyme inhibitor (ACEI), angiotensin receptor blocker (ARB), beta-blockers (BB), calcium channel blockers (CCB), and diuretics [29].

### BP and anthropometric measurements

All participants, after resting in a seated position for at least five minutes, systolic blood pressure (SBP) and diastolic blood pressure (DBP) were measured from the right arm by OMRON HBP-1300 (OMRON Healthcare Co., Ltd., Kyoto, Japan), at 5-minute intervals. Three readings were taken, and the average of the three measurements was computed for subsequent analysis. The height was measured with an RGZ-160 measurement device (Jiangsu Suhong Medical Instruments Co., Ltd., Jiangsu, China), and the weight of the participants wearing light clothing, without hats, shoes, coats, or any extra weights in the pockets, was measured using the InBody H20B measurement instrument (InBody Co., Ltd., Seoul, South Korea). Body mass index (BMI) was calculated by dividing a person’s weight (kg) divided by the square of their height (m) [41], and was categorized as underweight (BMI < 18.5 kg/m^2^), normal (BMI: 18.5–23.9 kg/m^2^), overweight (BMI: 23-24.9 kg/m^2^) and obese (BMI≥25 kg/m^2^), respectively.

### Blood sample collection and biomarker testing

After fasting for at least 8 h, 8ml of intravenous blood samples were collected for cryopreservation and sent to the Beijing ZhongtongLanbo laboratory (Beijing, China) to measure total cholesterol (TC), triglycerides (TG), HDL cholesterol (HDL-C), LDL cholesterol (LDL-C), fasting glucose and serum creatinine levels. In addition, urinary creatinine and albumin were measured using an enzymatic method and immunoturbidimetric assays to calculate the urinary albumin to creatinine (ACR) ratio. The estimated glomerular filtration rate (eGFR)was calculated by the following equation from “Chinese Modification of Diet in Renal Disease“ [42], eGFR = 175 × Scr^− 1.234^× age^− 0.179^ [if female, × 0.79].

### Target BP

Determining SBP and DBP as a cut-off for the study is done after revising many guidelines. In the past decade, the international guidelines and consensus reports have been continuously updated [43] with regard to targeting BP ranges with coexisting conditions and also at different age groups. BP < 140/90 for those aged < 80 [44], aged ≥ 80 targets at < 150/90 mmHg [45–46], for the age group < 60, it was < 140/90, and for the age group ≥ 60 yrs it was < 150/90 mmHg [47]. For underlying comorbid conditions, the recommendations were HTN with DM, the BP was targeted to < 140/85, CKD without overt proteinuria < 140/90, CKD with overt proteinuria < 130/90 mmHg [45], and HTN with DM or CKD the BP target was < 140/90 [47]. A target of SBP of < 120 mmHg [48] was recommended if the patients had at least one CVD risk factor. The proposed low BP objective in the Action to Control Cardiovascular Risk in Diabetes (ACCORD was not examined to the same extent in any other trials like the U.K. Prospective Diabetes Study (UKPDS), Hypertension Optimal Treatment (HOT), Systolic Hypertension in the Elderly Program (SHEP), and Systolic Hypertension in Europe (SYST-EUR) [49–50]. More detailed information on “Target BP” was provided in Supplementary file 3. There were many scoring systems that emerged to target SBP [51–52]. However, there are no such scoring systems or BP target guidelines for the Chinese population with comorbidities. Therefore, for all practical purposes, we considered a target BP of “140/90 mmHg” for hypertensive patients with or without comorbidities across the sex who were enrolled in our study.

### Comorbidity variables

Firstly, participants self-report their comorbidities by answering questions with a yes or no. After participants’ medical records are verified, qualified professionals check the data twice for relevant lab reports and prescriptions for any underlying chronic illnesses/ comorbidities to ensure its integrity and accuracy. We included only four comorbidities that are more prevalent in our study- dyslipidemia, CVD, CKD, DM. Patients with CVD were defined as having a history of hemorrhagic stroke, ischemic heart diseases, myocardial infarction, heart failure, and peripheral artery disease [7, 53]. According to the “Chinese Guidelines for the Management of Dyslipidemia (2016 edition)“ [54], the presence of one or more of the following was defined as dyslipidemia: high TG (≥ 2.26 mmol/L), high TC (≥ 6.22 mmol/L), high LDL-C (≥ 4.14 mmol/L), low HDL-C (< 1.04 mmol/L), or self-report on taking an anti-hypolipidemic drug. DM was defined as 7.0 mmol/L of fasting blood glucose or self-reported use of antidiabetic medications in the past two weeks, according to the Chinese Diabetes Society definition [55]. In addition, CKD was defined as the presence of albuminuria (UACR ≥ 30 mg/g) or eGFR < 60 ml/min/1.73 m^2^ [56].

### Statistical analyses

Descriptive statistics for continuous data were presented by mean, standard deviation, and t-test. Categorical variables were described by numbers and percentages (%), and intergroup comparisons were made using the Chi-square test. We compared comorbidities detection rates in subgroups stratified by age group and sex. To explore the potential effect of lifestyle modification and anti-hypertensive medication adherence instruction on BP control, we compared patients enrolled or not in BPHS management.

To further visualize the graphs and to construct the models, both the BPHS and non-BPHS groups were stratified according to the specific comorbidities (dyslipidemia, CVD, CKD, DM) and the number of the presence of the above-accumulated comorbidities (classified as with “comorbidity”, “without comorbidity”, “one comorbidity”, “two comorbidities”, and “≥ three comorbidities”). We used the binary logistic regression models to test the association of enrolled BPHS management and BP control, with BP control as the dependent variable (uncontrolled as the reference). Model 1 was adjusted for the age (35–44, 45–54, 55–64, and ≥ 65), sex (male vs. female), residence (urban residence vs. rural residence), nationality (Han minority vs. ethnic minorities), education attainment (primary school or lower vs. middle school or higher), family income (low income vs. high income). Model 2 added adjusted current smokers (yes vs. no), current drinkers (yes vs. no), and BMI (< 23 vs. ≥ 23). Model 3 was further adjusted for the types of anti-hypertensive medications taken. The odds ratio (OR) values and 95% confidence interval (CI) were calculated and presented in the forest plot, and all models were analyzed for multicollinearity to ensure their robustness. For the variables included in the models, the variance inflation factor (VIF) is less than 5, and there is no evidence of collinearity between the independent variables. To further elucidate sex differences in BP control rates, we constructed maps to visualize control rates among male and female participants at eight survey centers. Where relevant, Strengthening the Reporting of Observational Studies in Epidemiology (STROBE) guidelines reporting was followed (Supplementary file 4). All model goodness-of-fit, coefficients, variability, and models’ selection were provided with details in Supplementary file 5.

All statistical analyses were performed with IBM SPSS 20.0 software and R version 4.0.5 (https://www.r-project.org). *p* < 0.05 was considered statistically significant.

## Results

### General characteristics of the participants

A total of 1521 patients with previously known status of HTN, an average age of 62.6 ± 12.6 years, mainly being women (51.5%), living in urban areas (56.4%), Han (73.5%), and having a low income (72.6%) and low education level (59.0%), were included. Furthermore, 70.7% of patients (95% CI: 66.3-76.8%) had at least one coexisting comorbidity.

Among male and female hypertensive patients, 61.7% and 70.9% were managed by BPHS, respectively; most of them were 65 and older, as well as those with low household income and little education (untrained or under primary school but could read and write), which constituted the BPHS categories for both sex.

In the male BPHS group, the proportion of patients with comorbidities was 70.3%, and the proportion of those with three or more comorbidities (9.2%) was higher than in the non-BPHS group (5.0%). In the female BPHS group, 72.3% of patients had other coexisting diseases, which was significantly higher than that in the non-BPHS group (64.0%, *p* < 0.05), and the proportion of those with two (22.3%) and three or more comorbidities (6.8%) was slightly lower than that in the non-BPHS group (Table 1). Regardless of the comorbidity, both male and female hypertensive patients were more likely to have these coexisting conditions at 65 years and older (Table 2). But women had a higher prevalence of comorbidity than men (Dyslipidemia 51.3% vs. 35.2%, CVD 79.4% vs. 56.5%, CKD 57.1% vs. 49.8%, DM 50.4% vs. 49.2%).

**Table 1:** Comparison of the characteristics of HTN patients under BPHS or non-BPHS groups stratified by sex in Yunnan, China

Characteristics	Total (n = 1521)		Male (n = 737)				Female (n = 784)		
			BPHS (n = 455)	non-BPHS (n = 282)	*p*-value		BPHS (n = 556)	non-BPHS (n = 228)	*p*-value
**Age (years)**									
35–44	134 (8.8)		29 (6.4)	62 (22.0)	< 0.001		23 (4.1)	20 (8.8)	< 0.001
45–54	324 (21.3)		86 (18.9)	76 (27.0)			93 (16.7)	69 (30.3)	
55–64	365 (24.0)		94 (20.7)	70 (24.8)			139 (25.0)	62 (27.2)	
≥ 65	698 (45.9)		246 (54.1)	74 (26.2)			301 (54.1)	77 (33.8)	
**Urban residence**	858(56.4)		243 (53.4)	160 (56.7)	0.377		315 (56.7)	140 (61.4)	0.221
**Han people**	1118 (73.5)		319 (70.1)	211 (74.8)	0.166		397 (71.4)	191 (83.8)	< 0.001
**Low household income** ^**#**^	1104 (72.6)		324 (71.2)	166 (58.9)	0.001		447 (80.4)	167 (73.2)	0.027
**Education attainment, Primary school or below**	898(59.0)		224 (49.2)	101 (35.8)	< 0.001		430 (77.3)	143 (62.7)	< 0.001
**Current smokers**	223(14.7)		135 (29.7)	87 (30.9)	0.734		0 (0.0)	1 (0.4)	0.291
**Current drinkers**	267(17.6)		158 (34.7)	83 (29.4)	0.137		19 (3.4)	7 (3.1)	0.805
**BMI≥ 23 kg/m** ^**2**^	918(60.4)		312 (74.3)	195 (79.3)	0.145		382 (74.6)	149 (73.4)	0.739
**Comorbidity**									
Yes	1076 (70.7)		320 (70.3)	208 (73.8)	0.315		402 (72.3)	146 (64.0)	0.022
No	445 (29.3)		135 (29.7)	74 (26.2)			154 (27.7)	82 (36.0)	
**Number of comorbidities**									
0	445 (29.2)		135 (29.7)	74 (26.2)	< 0.001		154 (28.2)	82 (26.3)	0.023
1	634 (41.7)		169 (37.1)	118 (41.8)			244 (42.6)	103 (42.1)	
2	345 (22.7)		109 (24.0)	76 (27.0)			126 (22.3)	34 (23.7)	
≥3	97 (6.4)		42 (9.2)	14 (5.0)			32 (6.8)	9 (7.9)	

Note: Values are expressed as number (percentage); # Indicates household income < 4487 USD/year

Abbreviations: HTN hypertension, BPHS basic public health services, BMI body mass index

**Table 2:** Distribution of the four comorbidities in patients of different age groups

	Total†		Male#					Female#			
			35–44	45–54	55–64	≥ 65		35–44	45–54	55–64	≥ 65
Dyslipidemia	711 (46.7)		63 (8.9)	99 (13.9)	75 (10.6)	129 (18.1)		17 (2.4)	62 (8.7)	89 (12.5)	177 (24.9)
CVD	80 (5.3)		1 (1.2)	4 (5.0)	15 (18.8)	26 (32.5)		0 (0.0)	1 (1.2)	6 (7.5)	27 (33.8)
CKD	577 (37.9)		37 (6.4)	58 (10.0)	53 (9.2)	147 (25.5)		13 (2.3)	43 (7.4)	65 (11.3)	161 (27.9)
DM	259 (17.0)		15 (5.8)	21 (8.1)	29 (11.2)	63 (24.3)		6 (2.3)	19 (7.3)	40 (15.4)	66 (25.5)

†Numbers represent the proportion of those with comorbidities out of a total of 1521 hypertensive patients. Since a patient may have more than one comorbidity, the column total percentages are greater than 100%

# Numbers are presented as row percentages

### Lifestyle modification services in BPHS vs. non-BPHS groups

Male patients in the BPHS group were more likely to receive lifestyle modification and medication guidance, including reduced salt intake (89.6% vs. non-BPHS 82.6%), quit smoking (87.4% vs. 81.6%), reduced alcohol intake (87.8% vs. 81.5%), stress reduction guidance (81.8% vs. 73.7%), and were more likely to take anti-hypertensive medications (*p* < 0.05). Female participants were more likely to seek advice on improving physical activity and weight loss management compared to men (Table 3).

**Table 3:** Comparison of lifestyle modification guidance and medication taking under BPHS or non-BPHS groups stratified by sex

	Male (n = 737)					Female (n = 784)		
	BPHS	non-BPHS		*p*-value		BPHS	non-BPHS	*p*-value
**Lifestyle modification**								
Reduced sodium intake	407 (89.6)	232 (82.6)	0.006			494 (89.3)	184 (80.7)	0.001
Increased physical activity	386 (86.0)	225 (80.6)	0.057			485 (87.7)	175 (77.4)	< 0.001
Weight loss	372 (84.7)	221 (79.8)	0.087			450 (84.4)	165 (74.3)	0.001
Tobacco cessation	380 (87.4)	222 (81.6)	0.037			422 (80.8)	162 (75.0)	0.076
Reduced alcohol intake	381 (87.8)	225 (81.5)	0.021			423 (81.0)	161 (74.2)	0.037
Stress reduction intervention	359 (81.8)	196 (73.7)	0.011			422 (80.8)	154 (72.0)	0.008
**Number of BP-lowering medications taking**								
0	130 (28.6)	165 (58.5)	< 0.001			157 (28.3)	111 (48.7)	< 0.001
1	227 (49.9)	88 (31.2)				322 (57.9)	102 (44.7)	
2	82 (18.0)	26 (9.2)				69 (12.4)	15 (6.6)	
3	16 (3.5)	3 (1.1)				8 (1.4)	0 (0.0)	

Note: Values are expressed as number (percentage)

### BP control among individuals with comorbidities

**Fig. 1:**
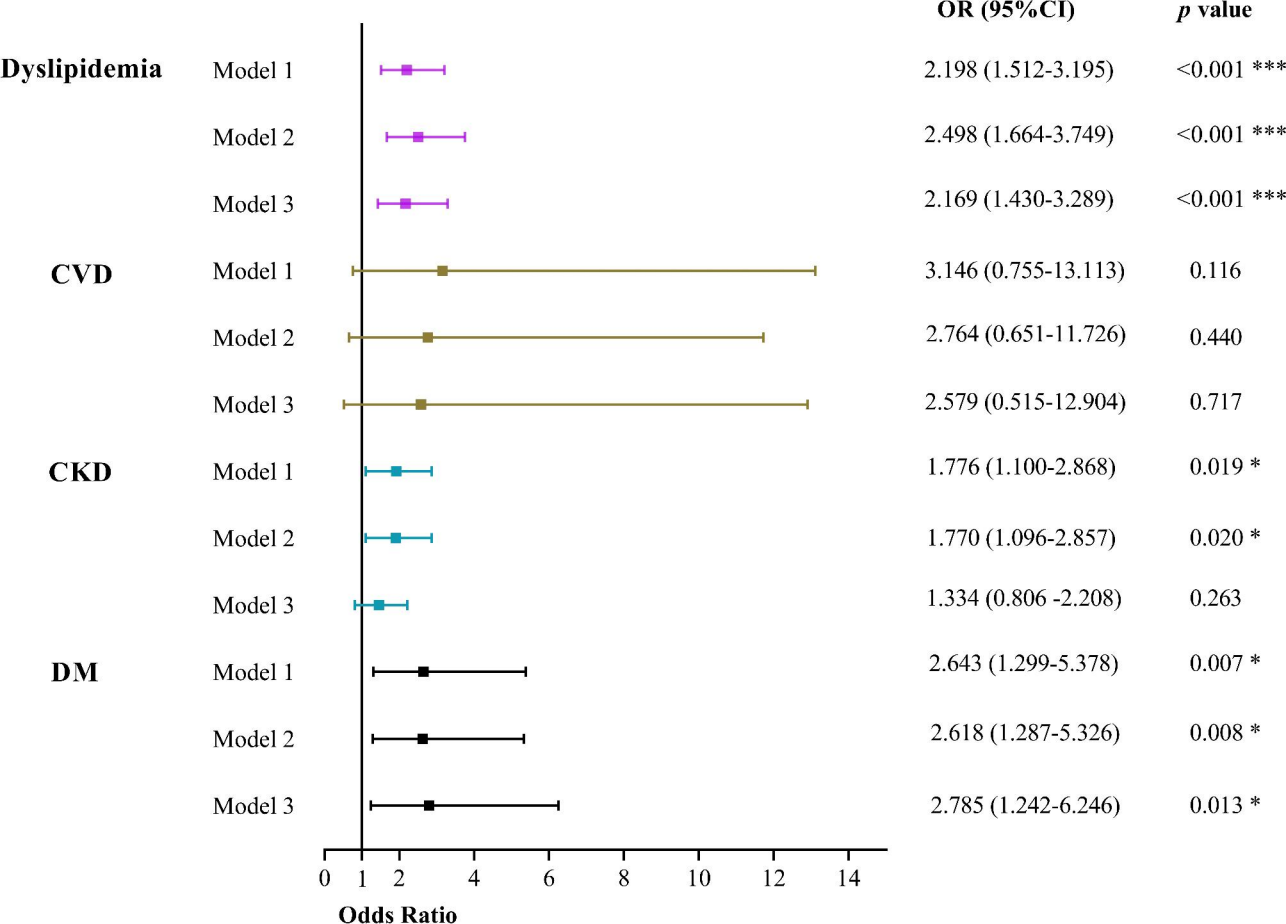
Association between BPHS management and BP control in patients with different comorbidities according to multivariate logistic regression models. Model 1 was adjusted for age, sex, residence, ethnicity, education level, and household income. Model 2 added adjusted smoke, drinking, and BMI based on Model 1. Model 3 further added adjusted kinds of anti-hypertensive medications. *Indicates p < 0.05, **indicates p < 0.01, *** indicates p < 0.001

Figure 1 shows that, using multivariable logistic regression models adjusted for confounders, it was demonstrated that BPHS maintained to promote BP control in patients with dyslipidemia (OR = 2.169, 95% CI: 1.430–3.289) and DM (OR = 2.785, 95%, CI: 1.242–6.246), both *p* < 0.05. However, the fully adjusted model was not statistically significant (*p* > 0.05) in HTN patients combined with CKD, as shown in Fig. 1 and Supplementary file 5.

**Fig. 2:**
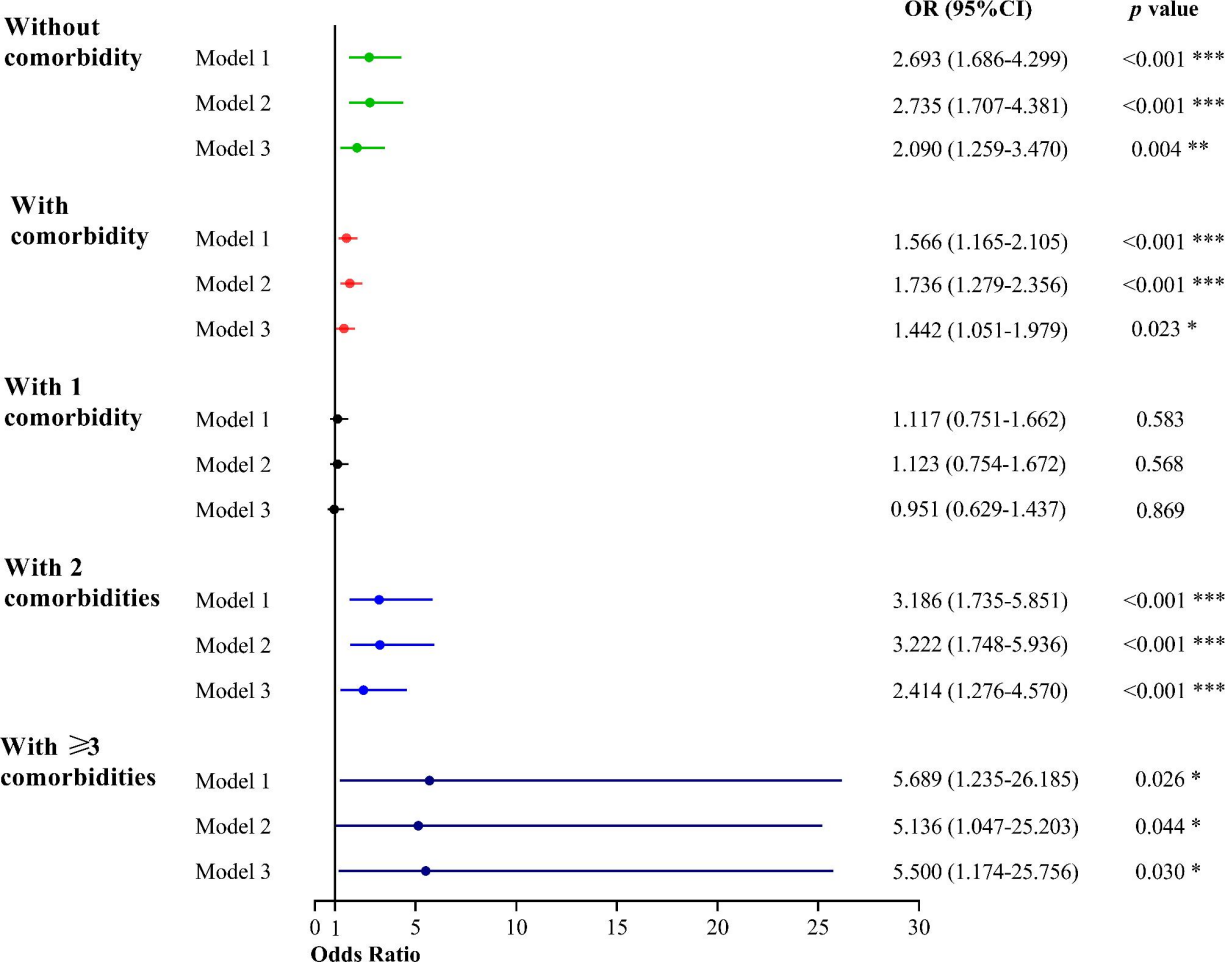
Association between BPHS management and BP control in patients with accumulated comorbidities according to multivariate logistic regression models. Model 1 was adjusted for age, sex, residence, ethnicity, education level, and household income. Model 2 added adjusted smoke, drinking, and BMI based on Model 1. Model 3 further added adjusted kinds of anti-hypertensive medications. *Indicates p < 0.05, **indicates p < 0.01, *** indicates p < 0.001

Figure 2 shows the association between BPHS (stratified by individuals without comorbidities compared to individuals with one / two / three comorbidities and BP control. In the 445 patients without comorbidities, BPHS demonstrated that it was beneficial to achieve BP control (OR = 2.090, 95%CI: 1.256–3.470). Among 1076 patients with comorbidities, a higher BP control was also observed in the BPHS group (OR = 1.442, 95% CI: 1.051–1.979). Furthermore, the BPHS group showed significantly better BP control among the patients with two comorbidities (OR = 2.414, 95% CI: 1.276–4.570) and three or more (OR = 5.500, 95%CI: 1.174–25.756).

### SBP/DBP variations in individuals with comorbidities across sex

**Fig. 3:**
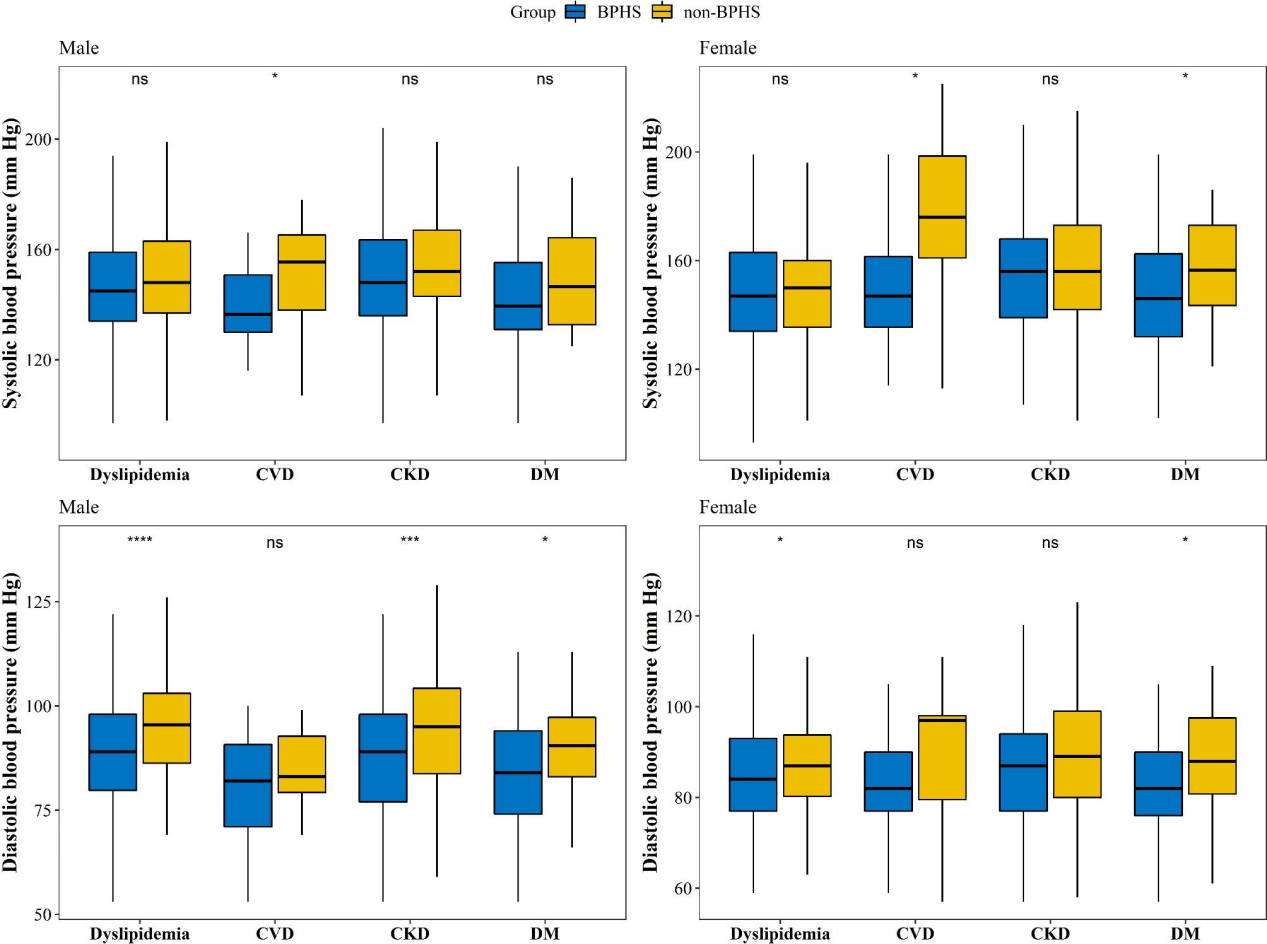
SBP/DBP difference with individual comorbidities under BPHS and non-BPHS management in Yunnan, China

Figure 3 shows the SBP and DBP in patients with four comorbidities in the BPHS and non-BPHS groups stratified by sex. Among male participants, SBP was significantly lower in the BPHS group in the presence of CVD compared to the non-BPHS group, while DBP was lower in patients with dyslipidemia, CKD, and DM (both *p < 0.05*). Both SBP and DBP were lower in female patients with DM in the BPHS group. Women with CVD in the BPHS group had lower SBP, and those with dyslipidemia had lower DBP(*p* < 0.05).

**Fig. 4:**
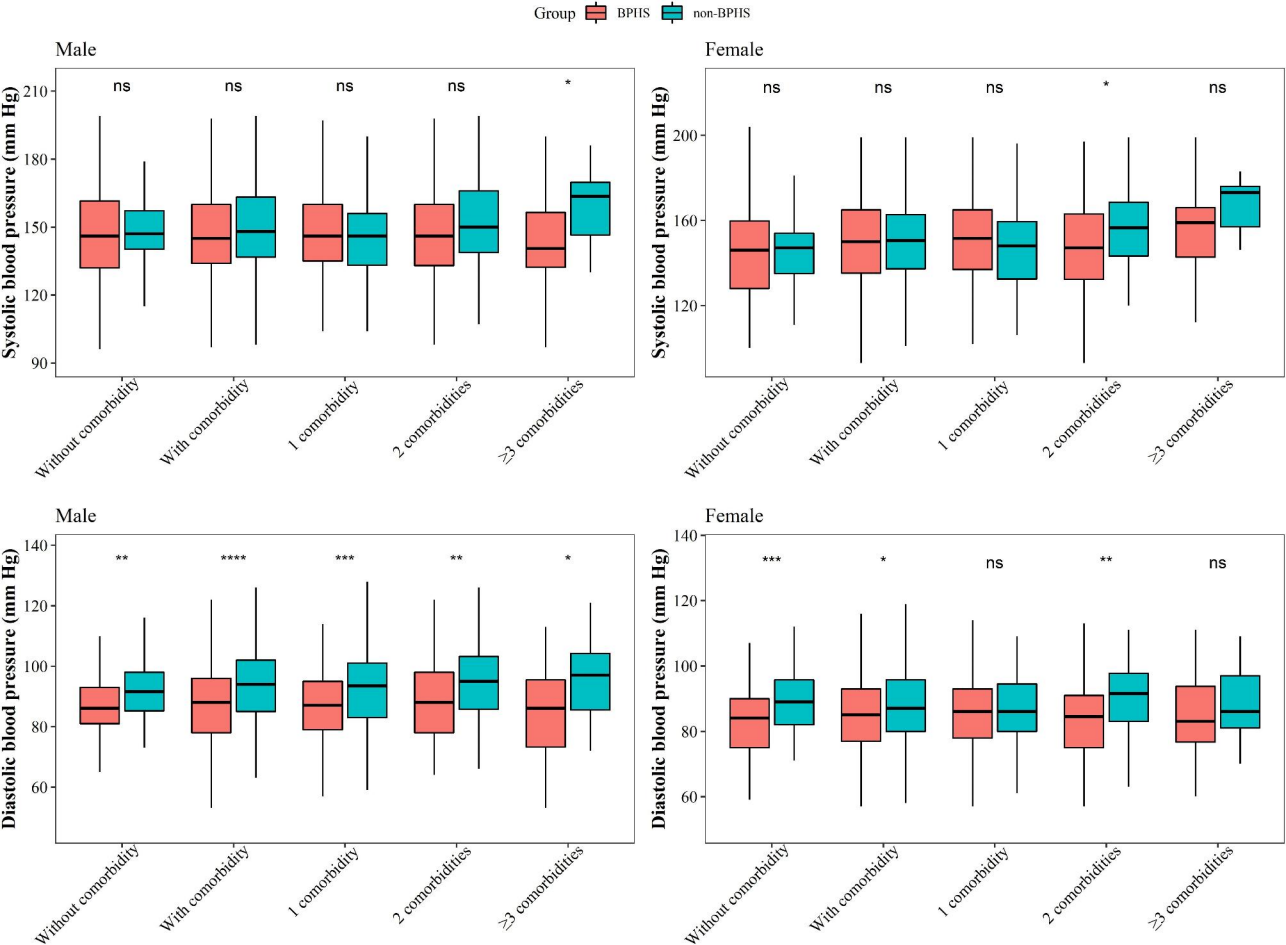
SBP/DBP difference with accumulated comorbidities under BPHS and non-BPHS management in Yunnan, China

Figure 4 presents the BP of the BPHS and non-BPHS groups of male and female patients with different numbers of comorbidities. Male HTN patients with three or more comorbidities in the BPHS group had significantly lower SBP than those in the non-BPHS group. DBP was lower regardless of the number of comorbidities. In female participants, SBP and DBP were lower in the BPHS group with two additional conditions.

### BP control among men and women from urban/rural communities

Participants were enrolled from both urban (Mengzi, Guandu, Dali, Zhaoyang) and rural (Dayao, Xinping, Chengjiang, Anning) survey centers in Yunnan Province. Overall, the BP control rate of male and female patients in the BPHS group was higher than that in the non-BPHS group from Dayao, Xinping, Mengzi, and Chengjiang. However, BP control rates were significantly higher in men than women in the urban areas of “Guandu” as well as in the rural area of “Chengjiang” (Supplementary file 6).

Supplementary file 7 shows the BP control rate in men and women with coexisting comorbidities in all eight study centers. Both male and female participants with dyslipidemia, CVD, CKD, and DM experienced a higher rate of BP control in the BPHS group than in the non-BPHS group. Despite having four comorbidities, men from the urban areas of “Guandu” (Fig. 1G in Supplementary file 7) and “Mengzi” (Fig. 1D in Supplementary file 7) in the BPHS group had a higher control rate than female patients.

## Discussion

This study explored the effectiveness of BPHS policy in reducing HTN among those with pre-existing long-term comorbidities. Also, it revealed whether women and men benefited equally from BPHS policy. We presented the findings of a survey associated with the BPHS program to analyze BP control in hypertensive patients with comorbidities. We found that 70.7% (1076/1521) of HTN patients had at least one coexisting comorbidity, which was higher than the percentage found in studies from Hong Kong (47.4%) [57] and the UK (51.0%) [58].

Dyslipidemia was the primary comorbidity for the majority of the participants (43.7%), followed by CKD (35.5%), which is consistent with the fact that dyslipidemia was the most prevalent comorbidity in the Chinese hypertensive population (about 41.3%) [59–60]. HTN combined with hypercholesterolemia has become the most important risk factor for ischemic heart disease mortality among Chinese residents [61]. HTN has resulted in 32.75 million disability-adjusted life years in entire China [62]; hypertensive patients with low socioeconomic status, low health literacy, and having lower access to medical services [63] require considerable health resource input, especially in south-west China, where the population is aging in a higher rate. Attention was deemed necessary in the interim since China has the highest proportion of CKD patients in Asia (up to 159.8 million) [64].

ISH [7] and ACC guidelines [27] recommend lifestyle intervention for CVD prevention and non-pharmacological treatment of hypertensive comorbidities as an effective means to lower BP. Consistent with ISH and ACC guidelines, the Chinese national standards for BPHS (the third edition) [31] recommend primary healthcare providers to create personalized models of lifestyle modification for hypertensive patients, to supervise and track constantly, to provide patient counseling, and to encourage patients to follow the recommended lifestyle changes consistently. Thus, lifestyle modification was included as an essential component of managing hypertensive patients [28, 31, 65]. This study found that a higher percentage of male hypertensive patients with comorbidities in the BPHS group received services related to salt reduction instruction (BPHS 89.6% vs. non-BPHS 82.6%), alcohol consumption reduction (87.8% vs.81.5%), and stress reduction guidance (81.8% vs. 73.7%). It is observed that the more the primary healthcare practitioners provided health education and lifestyle modifications to patients, the more it enhanced health awareness, helped in BP control, and encouraged people to adopt a better lifestyle [66–67].

This study also found that BPHS group patients with comorbidities had a higher proportion of taking a single drug (BPHS group 54.3% vs. non-BPHS group 37.2%) or combination of drugs (17.3% vs. 8.6%) than those in the non-BPHS group, which is in line with a report stating that HTN patients with comorbidities typically took more medications for a longer period of time to manage their blood pressure [7, 29]. ‘Consistency’ could be the key to effectively controlling BP in comorbid patients in the BPHS group. A patient-centered approach to clinical practice and the use of herbal medications were linked to optimum BP control in earlier studies, which may offer guidance for future BPHS policy amendments [68–69].

Moreover, our study observed lower DBP and SBP in the BPHS group, regardless of the different types of comorbidities or accumulated comorbidities. As hypertensive dyslipidemia or DM, patients were 2.169 times and 2.785 times more likely to receive BPHS management, respectively, and a similar trend was observed in patients with comorbidities clusters. The above findings further provided sufficient evidence that BPHS can effectively manage HTN with comorbidities in the low-income provinces of south-west China. However, the adjusted model from HTN patients with CKD was not statistically significant (p > 0.05), and the results imply that “anti-hypertensive medication” appears to be a substantial and important predictor of BP control, which detracts from/influences the potential association between the independent variable (BPHS management) and the dependent variable (BP control). It is evident that the BPHS management system should be a priority consideration for hypertensive patients with comorbid CKD. Unlike the current clinical practice recommendations, which primarily address managing HTN from a single comorbidity [70], our findings support the trend among primary healthcare providers to monitor BP and assess risks to manage the targeted BP better and provide high-quality services, as per international guidelines for the treatment of hypertensive patients with multiple and accumulated comorbidities.

In addition, this study demonstrated that, compared to the non-BPHS group, the BPHS care group was beneficial in lowering SBP and DBP with multiple comorbidities in both sex. In some high-income urban survey centers, men appeared more likely to have their BP well under control. Previous studies from Denmark and the United States have reported differences in BP control rates depending on the presence of different diseases in men as compared to women [53, 71]. Current HTN recommendations give sex disparity relatively minimal consideration [23, 72]. Therefore, national health initiatives like BPHS must consider this inequality. This can be achieved by providing additional BPHS resources, particularly in areas with limited access to female patients with multiple comorbidities.

The study reveals that BPHS would significantly contribute to the ‘targeted BP control’ among patients with comorbidities and encourage them to adhere to BPHS-assisted lifestyle changes. Furthermore, by imparting the most recent information and experience, it would benefit prospective policies in LMIC regions.

### Limitation

The results could have been impacted by the duration and occurrence of CKD, CVD, and DM, but we did not validate this interdependency in this study. The relationship between BPHS management and BP control should be evaluated cautiously due to the limitations imposed by the cross-sectional study methodology. Secondly, the efficiency of HTN management could have been unintentionally underestimated in hypertensive patients with comorbidities (1, 2, 3, or more comorbidities) owing to the short duration of BPHS services provided. Thirdly, this study identified four comorbidities as the most prevalent comorbidities (dyslipidemia, DM, CKD, CVD) in the Chinese populace, and thus only these four were considered in inclusion criteria, and minor comorbidities like Gout, rheumatic changes, dementia, and tumors were excluded from the study. Due to a very small sample size of patients in the group with four major comorbidities in some survey centers attributed to a lack of ‘scientific literacy’ in general, therefore both BPHS and non-BPHS enrollment trajectories declined significantly. This is inexorably shown on individual study center maps, which presents as extreme values of cases with control rates of “0 “or “100”, especially “Anning” study center (see Supplementary file 7).

**Our recommendations to health policymakers are**: (a) Future prospective cohort studies should consider enrolling a larger sample size, as doing so could help to better assess the effectiveness of BPHS in lowering BP, reducing related disability, and preserving medical and health resources in hypertension patients with complications. (b) NCD’s awareness camps should be conducted prior to study, as centers like “Anning” seems to lack ‘scientific literacy’ in general. (c) Furthermore, an emphasis on extensive research is necessary to comprehend the mechanisms by which sex influences the onset of HTN and vascular aging, as well as how this correlation can help in the early prevention of other comorbid conditions. (d) Due to the constant changes in the guidelines [73] for treating HTN, there should be an introduction of Chinese HTN education program recommendations for the management of hypertension, which should be taught to healthcare providers on a regular basis. (e) By collaborating with other Asian societies of HTN [74–75] and sharing knowledge with international committees working on HTN, it is essential to develop a HTN treatment specifically tailored to suit the Chinese race.

## Conclusion

This study evaluated the community-based Chinese BPHS program for effectively managing male and female hypertensive patients with comorbidities and found that almost two-thirds of the hypertensive patients have comorbidity in Yunnan Province. The BPHS program also successfully encouraged healthy lifestyle changes, lowered DBP and SBP, and improved BP control in HTN patients with various comorbidities. However, we also observed that male patients with HTN seemed to benefit more from BPHS than women. For the first time in China, this study results call for better management strategies and allocation of health care resources for chronic diseases, especially to women in older age group.

### Electronic supplementary material

Below is the link to the electronic supplementary material.

Supplementary Material 1

Supplementary Material 2

Supplementary Material 3

Supplementary Material 4

Supplementary Material 5

Supplementary Material 6

Supplementary Material 7

## Data Availability

The data supporting the findings of this study and its supplementary material are available on reasonable request from the project administrator/principal corresponding author (LD).

## Abbreviations

BMI: Body mass index
BP: Blood pressure
BPHS: Basic public health services
CI: Confidence interval
CKD: Chronic kidney disease
CVD: Cardiovascular diseases
DBP: Diastolic blood pressure
DM: Diabetes mellitus
HDL-C: High-density lipoprotein cholesterol
HTN: Hypertension
LDL-C: Low-density lipoprotein cholesterol
LMICs: Low and middle-income countries
NCD: Non-communicable diseases
OR: Odds ratio
SBP: Systolic blood pressure
TC: Total cholesterol
TG: Triglycerides
