# Supplementary material for: A tryst of ‘blood pressure control- sex- comorbidities’: the odyssey of basic public health services in Yunnan in quest for truth
Source: BMC Public Health. 2024 Feb 16;24:490. doi: 10.1186/s12889-023-17157-7 (PMC10870683; doi:10.1186/s12889-023-17157-7)
Supplement: Supplementary file 1 — Supplementary Material 1 [file 12889_2023_17157_MOESM1_ESM.docx]

**Supplementary file 1**

Figure 1. Sampling procedure of 9,600 participants in eight counties of Yunnan Province, PPS: probability proportional to size; SRS: simple random sampling

**129 counties/districts** in Yunnan Province

**Urban**

Stratified

**Rural**

Selected **4 cities** (namely, Guandu, Zhaoyang, Mengzi, Dali)

Selected **4 counties** (namely, Chengjiang, Anning, Xinping, Dayao)

PPS method

Selected **2 neighborhoods** in each city

Selected **2 townships** in each county

Selected **3 residential committees** in each neighborhood

Selected **3 villages** in each township

9600 participants aged ≥ 18 years

Selected **200 adults** in each residential committee

Selected **200 adults** in each village

SRS method

SRS method

SRS method

Figure 2. Inclusion process for 1521 study subjects

Patients 35 years and older diagnosed with hypertension (n=2764)

Study subjects who already knew they had hypertension (n=1521) ^#^

BPHS group (n=1011)

non-BPHS group (n=510)

Excluded:

- Missing ID number (n=83)
- Newly diagnosed *(n=1160)

With Comorbidity (n=722)

Without Comorbidity (n=289)

With Comorbidity (n=354)

Without Comorbidity (n=156)

9600 participants aged ≥ 18 years from Yunnan Province

* Indicated diagnosed hypertension at the time of data collection (blood pressure ≥140/90 mmHg).

# The Basic Public Health Services (BPHS) program in China requires primary care institutions to enroll patients 35 years of age and older who have been diagnosed with hypertension into the BPHS system, and to provide them with hypertension care and management by primary care providers at no cost. Accordingly, we included 1521 previously known hypertensive patients in this survey in the study with the aim of linking their unique identifiers to the BPHS system as a way to identify whether these study subjects were managed by the BPHS program.
