# Supplementary figures and images for: A tryst of ‘blood pressure control- sex- comorbidities’: the odyssey of basic public health services in Yunnan in quest for truth
Source: BMC Public Health. 2024 Feb 16;24:490. doi: 10.1186/s12889-023-17157-7 (PMC10870683; doi:10.1186/s12889-023-17157-7)

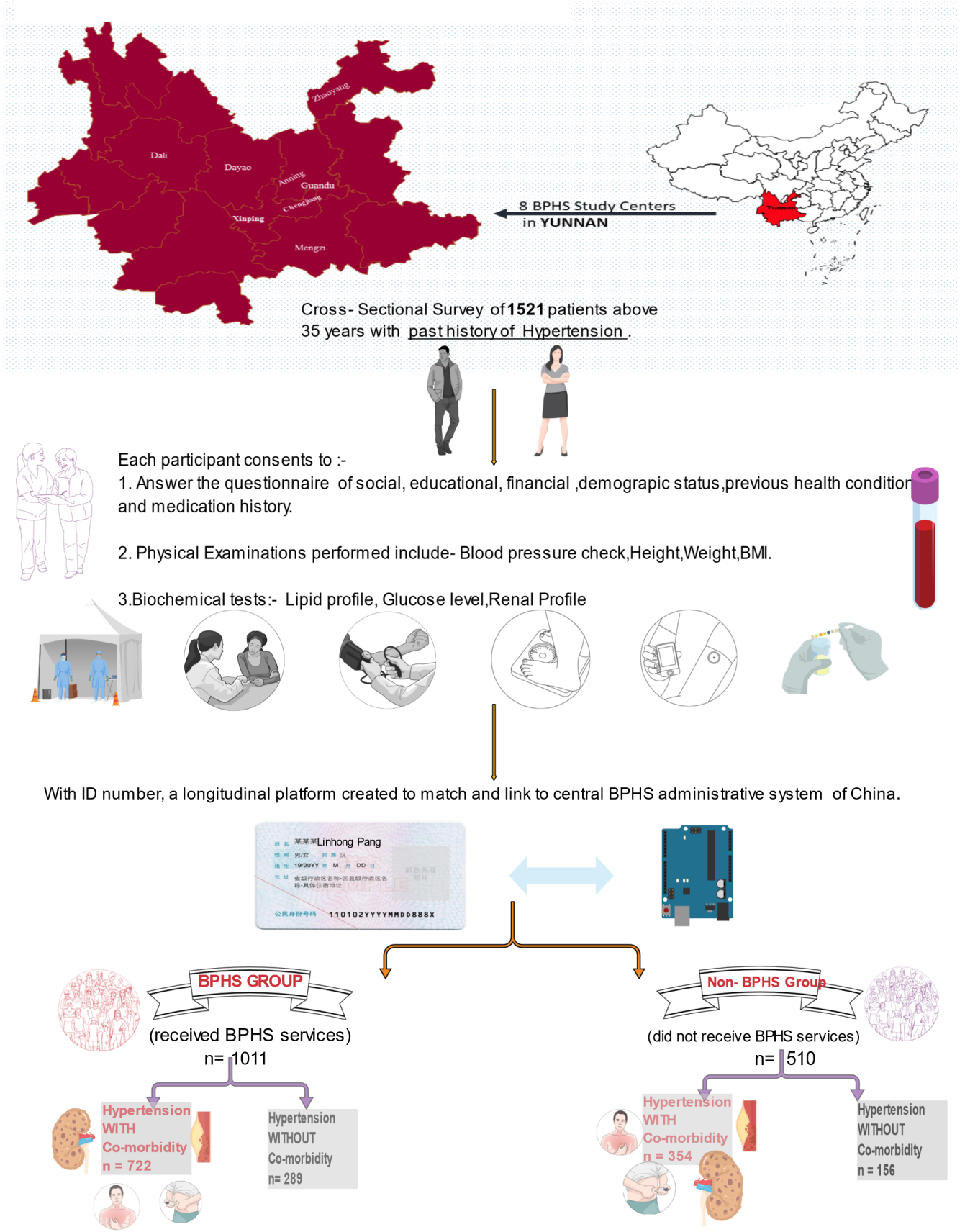

Supplement: Supplementary file 2 — Supplementary Material 2 [file 12889_2023_17157_MOESM2_ESM.pdf]

## Supplementary file 6

### Figure 1. Male- Female participants in each center with their 'BP control rate'

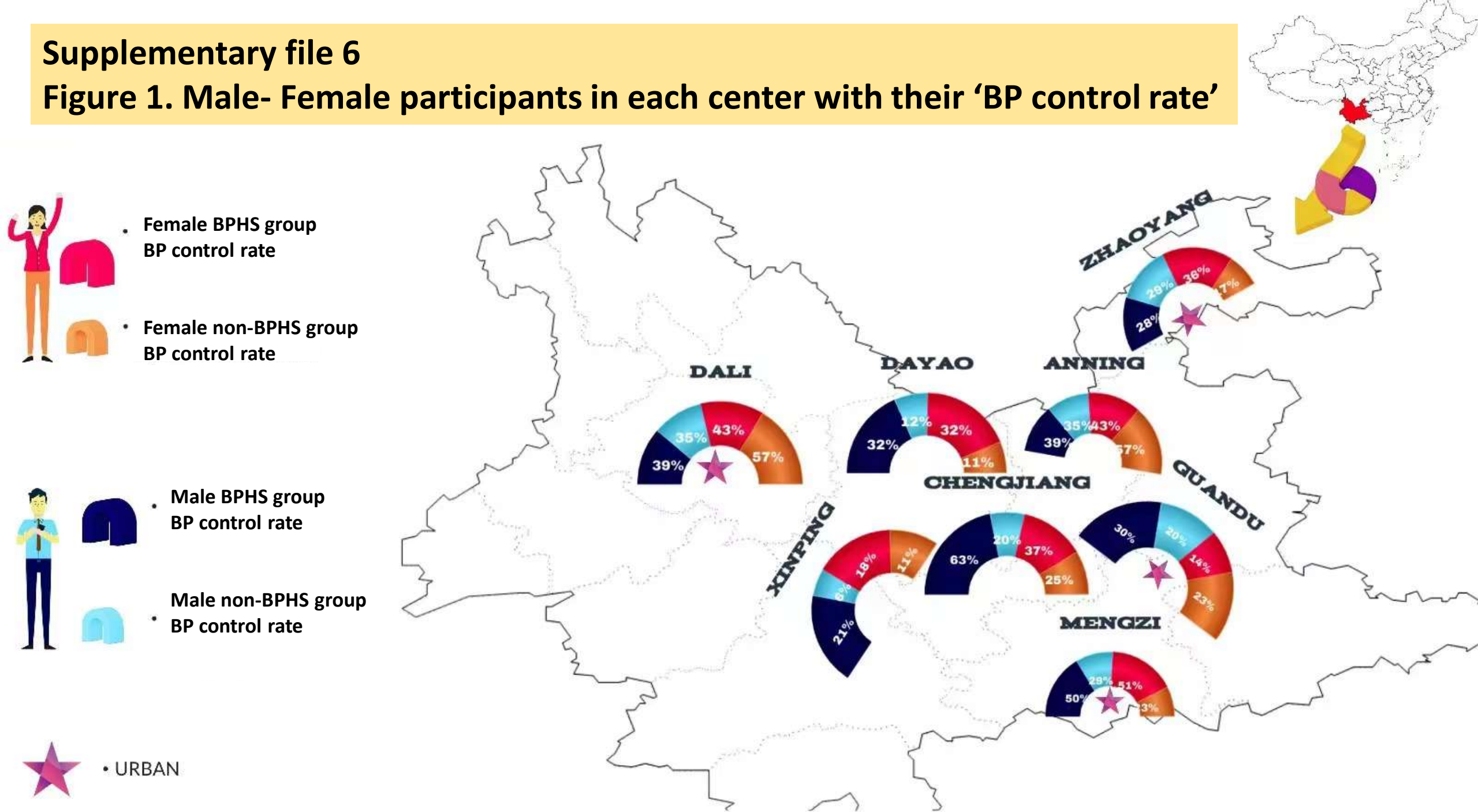

Supplement: Supplementary file 6 — Supplementary Material 6 [file 12889_2023_17157_MOESM6_ESM.pdf]

A

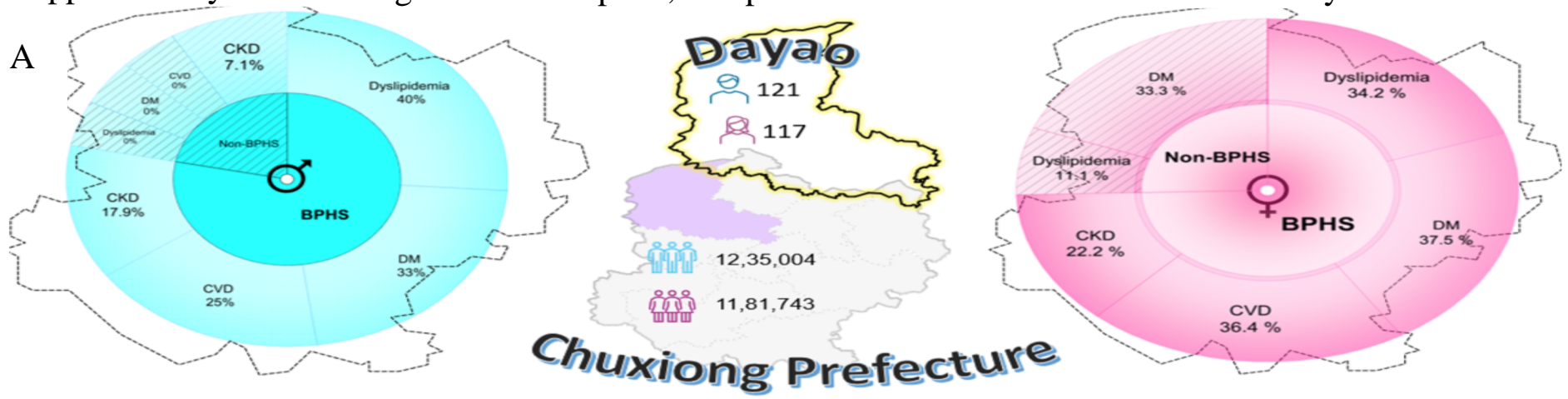

B

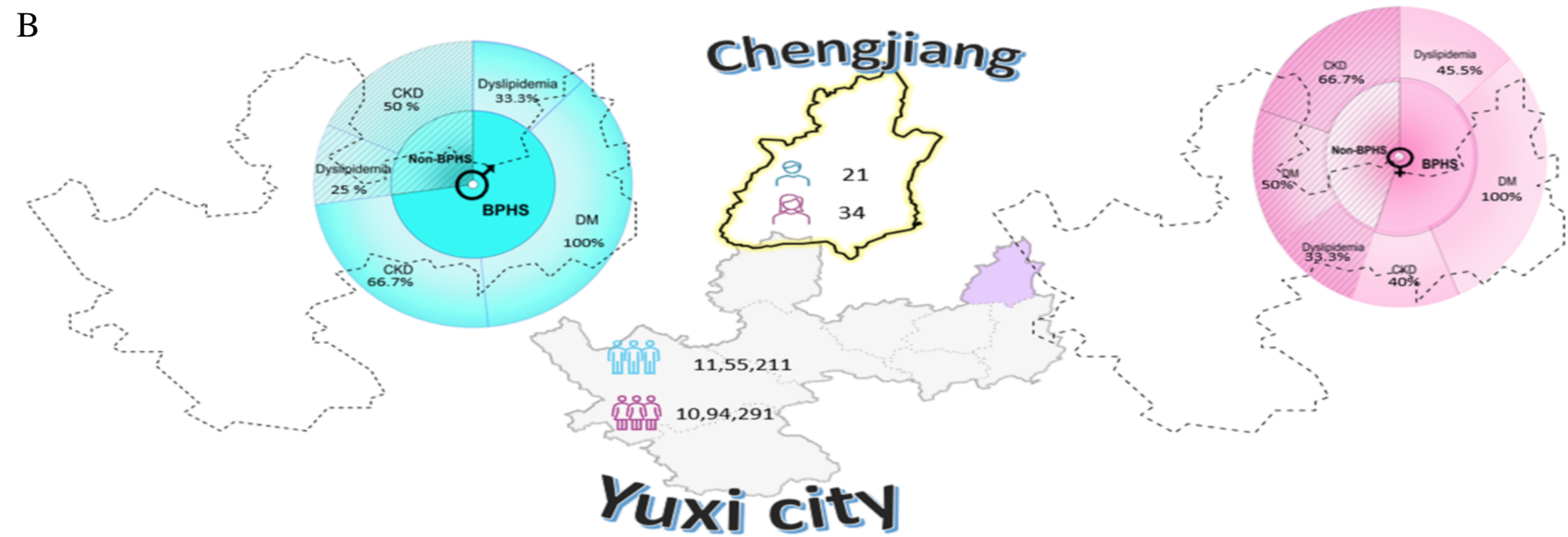

C

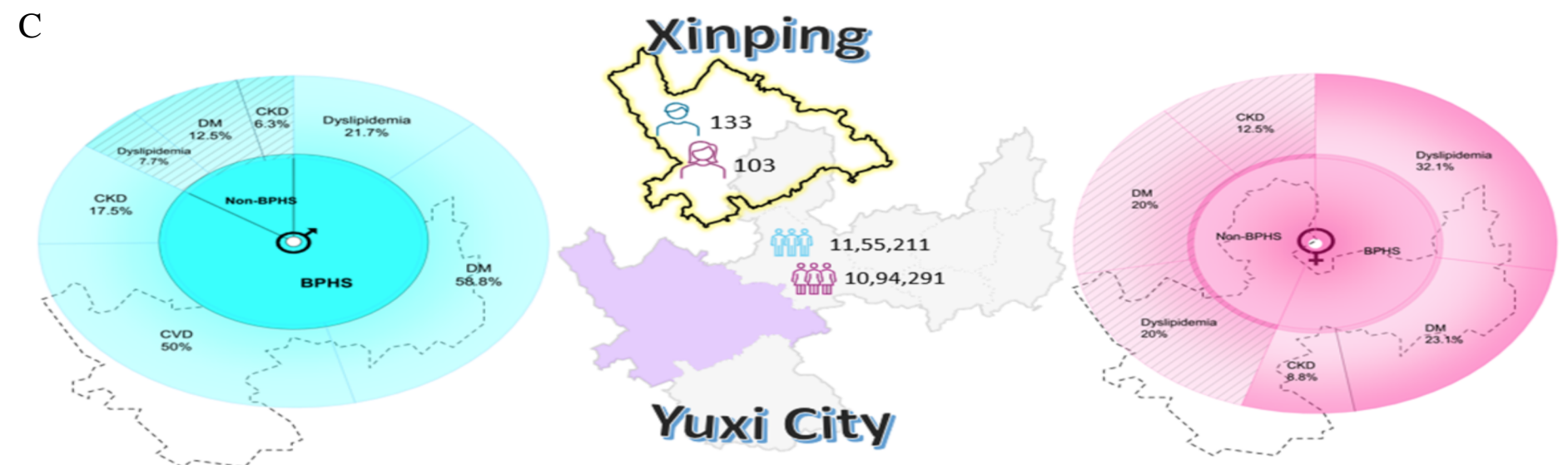

D

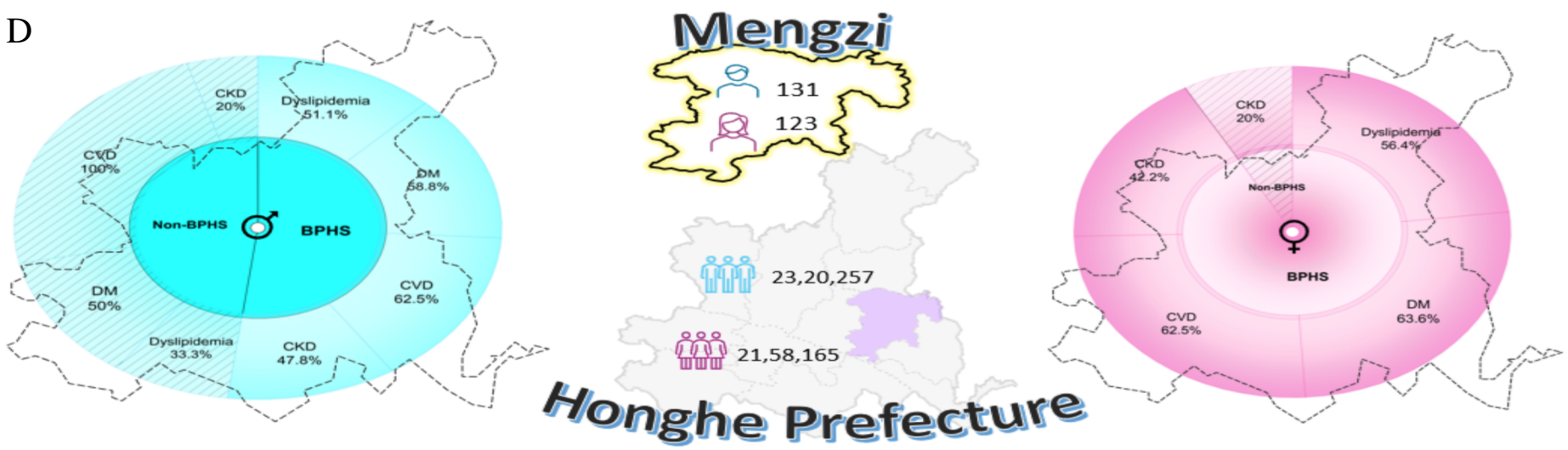

E

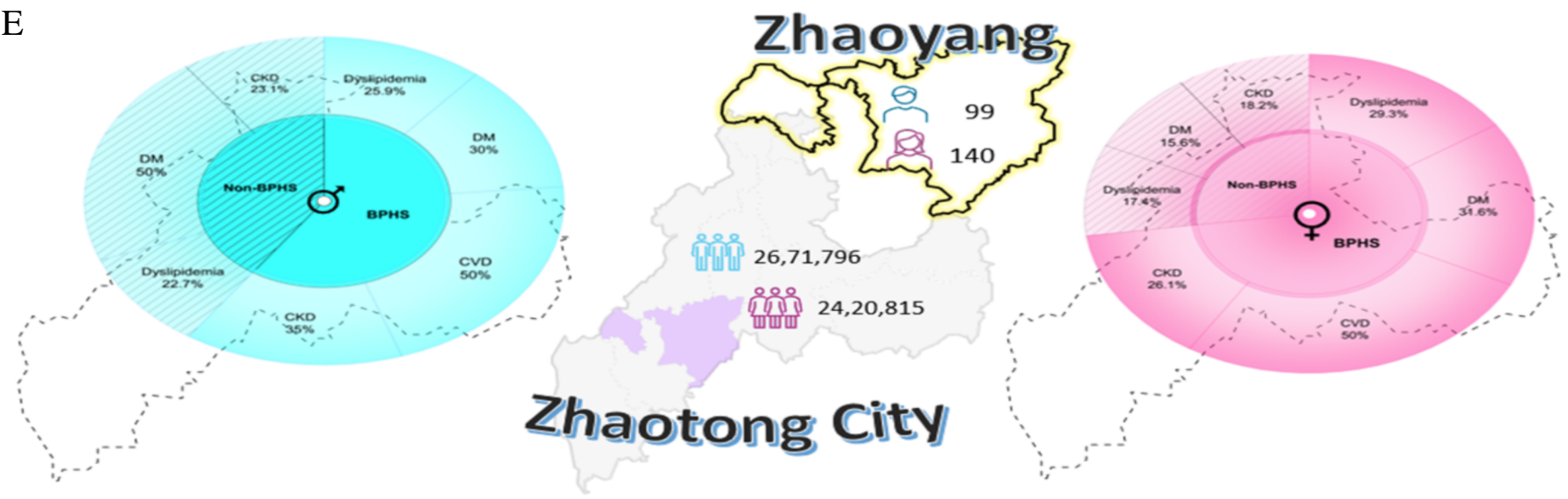

F

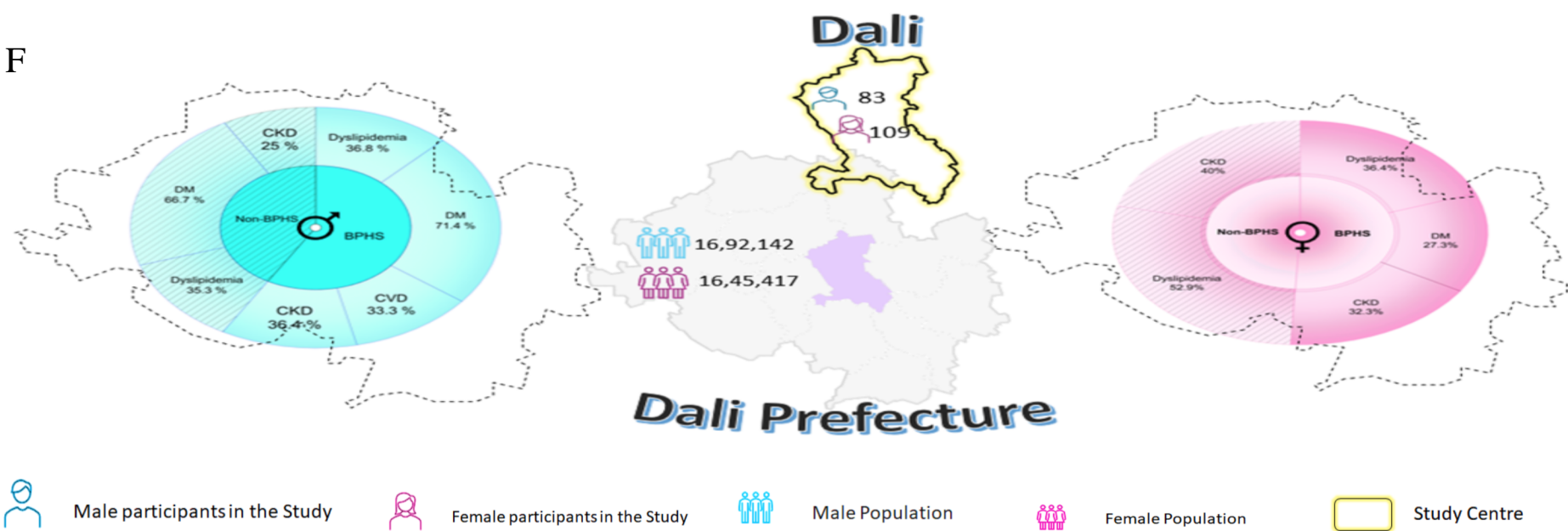

G

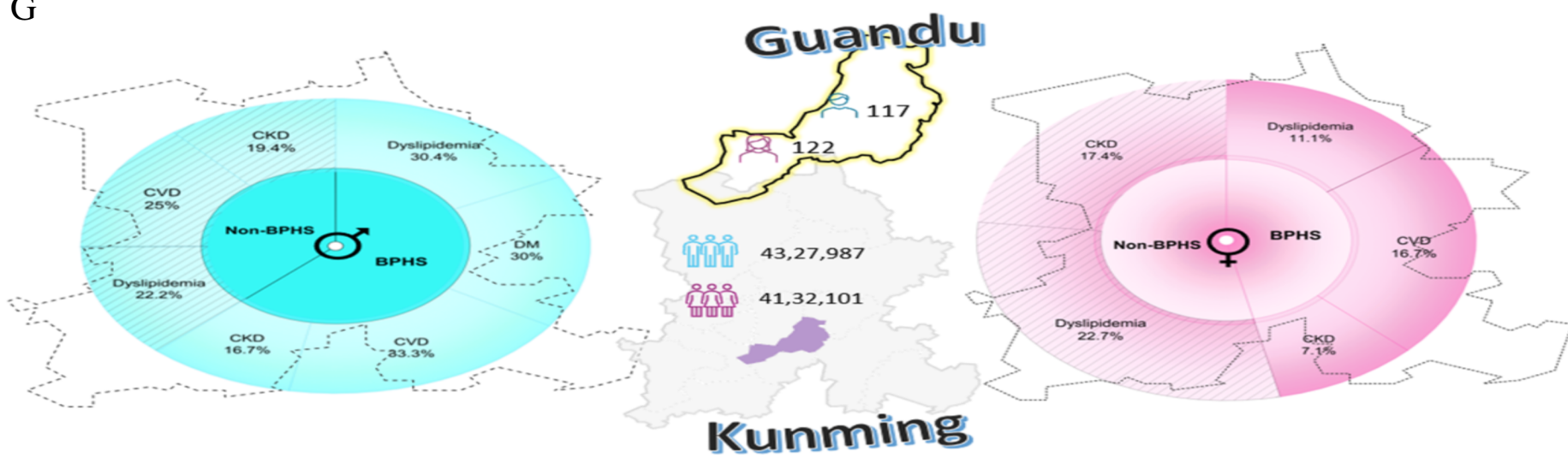

H

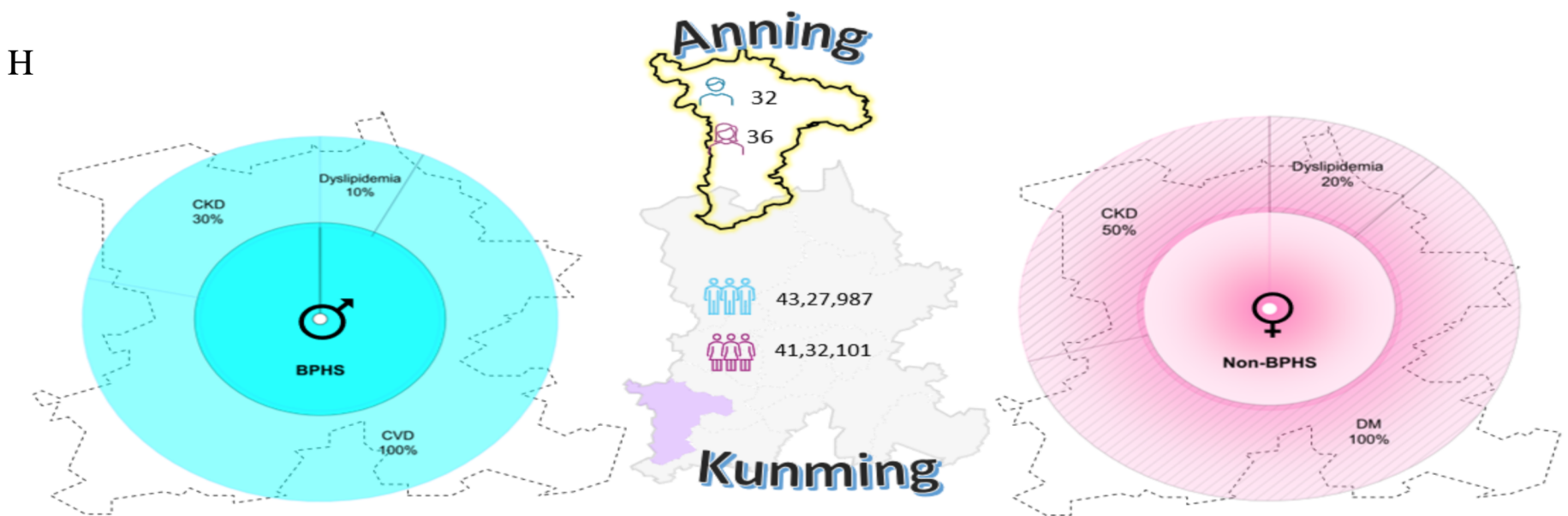

Supplement: Supplementary file 7 — Supplementary Material 7 [file 12889_2023_17157_MOESM7_ESM.pdf]
