# Supplementary material for: A tryst of ‘blood pressure control- sex- comorbidities’: the odyssey of basic public health services in Yunnan in quest for truth
Source: BMC Public Health. 2024 Feb 16;24:490. doi: 10.1186/s12889-023-17157-7 (PMC10870683; doi:10.1186/s12889-023-17157-7)
Supplement: Supplementary file 3 — Supplementary Material 3 [file 12889_2023_17157_MOESM3_ESM.docx]

**Supplementary file 3**

Table S1. "Target blood pressure" for different guidelines

| Blood-pressure goals according to current hypertension guidelines: - | | | |
| --- | --- | --- | --- |
|  | Guidelines  (Publication year) | Target Population | BP goal (mmHg) |
| 1 | NICE 2011 | Aged < 80years  Aged ≥ 80years | <140/90  <150/90 |
| 2 | EUROPEAN SOCIETY OF HYPERTENSION/ ESC 2013 | Aged < 80years  Aged> 80years  Diabetes mellitus  CKD without overt proteinuria  CKD with overt proteinuria | <140/90  <150/90  <140/85  <140/90  <130/90 |
| 3 | EIGHTH US JOINT NATIONAL COMMITTEE 2014 | Aged <60 years  Aged ≥60 years  Diabetes or CKD | <140/90  <150/90  <140/90 |
| 4 | AMERICAN SOCIETY OF HYPERTENSION/ INTERNATIONAL SOCIETY OF HYPERTENSION 2014 | Aged < 80years  Aged ≥ 80years | <140/90  <150/90 |
| Courtesy of table- Professor Brian Rayner(University of Cape town, South Africa)Ruiz-Hurtado G, Banegas JR, Sarafidis PA, Volpe M, Williams B, Ruilope LM. Has the SPRINT trial introduced a new blood-pressure goal in hypertension?. Nat Rev Cardiol. 2017;14(9):560-566. doi:10.1038/nrcardio.2017.74 | | | |
