# Supplementary material for: A tryst of ‘blood pressure control- sex- comorbidities’: the odyssey of basic public health services in Yunnan in quest for truth
Source: BMC Public Health. 2024 Feb 16;24:490. doi: 10.1186/s12889-023-17157-7 (PMC10870683; doi:10.1186/s12889-023-17157-7)
Supplement: Supplementary file 5 — Supplementary Material 5 [file 12889_2023_17157_MOESM5_ESM.docx]

**Supplementary file 5**

**Table S1. Model fitting parameters for Figure 1 in the manuscript (Details of models from Figure 1)**

|  | BP control rate, % | | Model 1 | | | | | | Model 2 | | | | | | Model 3 | | | | |  |
| --- | --- | --- | --- | --- | --- | --- | --- | --- | --- | --- | --- | --- | --- | --- | --- | --- | --- | --- | --- | --- |
|  | |  | **OR, 95%CI** | **R^2^** | **β** | **S.E.** | **Wald χ^2^** | **Intercept** | **OR, 95%CI** | **R^2^** | **β** | **S.E.** | **Wald χ^2^** | **Intercept** | **OR, 95%CI** | **R^2^** | **β** | **S.E.** | **Wald χ^2^** | **Intercept** |
| **Dyslipidemia (n=711)** | |  |  |  |  |  |  |  |  |  |  |  |  |  |  |  |  |  |  |  |
| non-BPHS | | 21 | 1 (Reference) | |  |  |  |  | 1 (Reference) | |  |  |  |  | 1 (Reference) | |  |  |  |  |
| BPHS | | 34 | 2.198 (1.512-3.195) *** | 0.061 | 0.788 | 0.191 | 17.028 | -1.97 | 2.498 (1.664-3.749) *** | 0.089 | 0.915 | 0.207 | 19.516 | -2.01 | 2.169 (1.430-3.289) *** | 0.096 | 0.774 | 0.212 | 13.271 | -2.46 |
| **CVD (n=80)** | |  |  |  |  |  |  |  |  |  |  |  |  |  |  |  |  |  |  |  |
| non-BPHS | | 23.5 | 1 (Reference) | |  |  |  |  | 1 (Reference) | |  |  |  |  | 1 (Reference) | |  |  |  |  |
| BPHS | | 41.3 | 3.146 (0.755-13.113) | 0.189 | 1.148 | 0.731 | 2.469 | -21.55 | 2.764 (0.651-11.726) | 0.214 | 1.026 | 0.738 | 1.935 | -22.832 | 2.579 (0.515-12.904) | 0.309 | 0.334 | 0.923 | 0.131 | -21.941 |
| **CKD (n=577)** | |  |  |  |  |  |  |  |  |  |  |  |  |  |  |  |  |  |  |  |
| non-BPHS | | 17.5 | 1 (Reference) | |  |  |  |  | 1 (Reference) | |  |  |  |  | 1 (Reference) | |  |  |  |  |
| BPHS | | 26.8 | 1.776 (1.100-2.868) * | 0.079 | 0.574 | 0.244 | 5.52 | -2.715 | 1.770 (1.096-2.857) * | 0.079 | 0.571 | 0.244 | 5.454 | -2.783 | 1.334 (0.806 -2.208) | 0.132 | 0.288 | 0.257 | 1.254 | -3.167 |
| **DM (n=259)** | |  |  |  |  |  |  |  |  |  |  |  |  |  |  |  |  |  |  |  |
| non-BPHS | | 20.6 | 1 (Reference) | |  |  |  |  | 1 (Reference) | |  |  |  |  | 1 (Reference) |  |  |  |  |  |
| BPHS | | 39.3 | 2.643 (1.299-5.378) ** | 0.081 | 0.972 | 0.363 | 7.186 | -1.197 | 2.618 (1.287-5.326) ** | 0.084 | 0.963 | 0.362 | 7.059 | -1.375 | 2.785 (1.242-6.246) * | 0.245 | 1.024 | 0.412 | 6.178 | -2.165 |

1 indicates that the non-BPHS group is taken as the reference group in the logistic regression model.

For the variables included in the models, the variance inflation factor (VIF) is less than 5, and there is no evidence of collinearity between the independent variables.

*Indicated p<0.05

**Indicated p<0.01

*** Indicated p<0.001

**Table S2. Model tests, selection of Figure 1 in the manuscript (Details of models from Figure 1)**

|  |  | Model 1 |  |  | Model 2 |  |  | Model 3 |  |
| --- | --- | --- | --- | --- | --- | --- | --- | --- | --- |
|  | **-2 log likelihood，**  **-2LL** | **Hosmer-Lemeshow tests, p** | **Omnibus Tests of Model Coefficients, p** | **-2 log likelihood，**  **-2LL** | **Hosmer-Lemeshow tests, p** | **Omnibus Tests of Model Coefficients, p** | **-2 log likelihood，**  **-2LL** | **Hosmer-Lemeshow tests, p** | **Omnibus Tests of Model Coefficients, p** |
| **Dyslipidemia** | 847.6 | - | <0.001 | 736.3 | 0.31 | <0.001 | 727.1 | 0.671 | <0.001 |
| **CVD** | 103.9 | - | 0.169 | 84.4 | 0.28 | 0.821 | 72.95 | 0.61 | 0.296 |
| **CKD** | 628.8 | - | 0.014 | 549.6 | 0.72 | 0.001 | 526.7 | 0.95 | <0.001 |
| **DM** | 325.1 | - | 0.004 | 275.8 | 0.64 | 0.048 | 261.9 | 0.69 | 0.002 |

The Hosmer-Lemesho goodness-of-fit test (H-L test) can be used to evaluate whether the model makes full use of the available information to maximize the fit of the model, explaining the variance of the model.

This study can indicate that the model fit superiority effect is good if P>0.05, and can model construction is poor if P<0.05.

The -2 log likelihood (-2LL) is an important index for model evaluation, the smaller the value, the better, which can be used for different models to evaluate the effect.

Model 1 did not adjust for any confounding variables, so p-values for the Hosmer-Lemeshow tests were not available.

**Table S3. Model fitting parameters for Figure 2 (Details of models from Figure 2)**

|  | BP control rate, % | Model 1 | | | | | | Model 2 | | | | | | Model 3 | | | | | |
| --- | --- | --- | --- | --- | --- | --- | --- | --- | --- | --- | --- | --- | --- | --- | --- | --- | --- | --- | --- |
|  |  | **OR, 95%CI** | **R^2^** | **β** | **S.E.** | **Wald χ^2^** | **Intercept** | **OR, 95%CI** | **R^2^** | **β** | **S.E.** | **Wald χ^2^** | **Intercept** | **OR, 95%CI** | **R^2^** | **β** | **S.E.** | **Wald χ^2^** | **Intercept** |
| **Without comorbidity (n=445)** | |  |  |  |  |  |  |  |  |  |  |  |  |  |  |  |  |  |  |
| non-BPHS | 21.2 | 1 (Reference) |  |  |  |  |  | 1 (Reference) |  |  |  |  |  | 1 (Reference) |  |  |  |  |  |
| BPHS | 38.8 | 2.693 (1.686-4.299) *** | 0.141 | 1.01 | 0.246 | 16.839 | -3.116 | 2.735 (1.707-4.381) *** | 0.169 | 1.036 | 0.265 | 15.273 | -3.097 | 2.09 (1.259-3.470) ** | 0.229 | 0.765 | 0.276 | 7.658 | -3.4 |
| **With comorbidity (n=1076)** | |  |  |  |  |  |  |  |  |  |  |  |  |  |  |  |  |  |  |
| non-BPHS | 22.3 | 1 (Reference) |  |  |  |  |  | 1 (Reference) |  |  |  |  |  | 1 (Reference) |  |  |  |  |  |
| BPHS | 31 | 1.566 (1.165-2.105) *** | 0.054 | 0.504 | 0.163 | 9.585 | -2.308 | 1.736 (1.279-2.356) *** | 0.047 | 0.552 | 0.156 | 12.548 | -1.846 | 1.442(1.051-1.979) * | 0.09 | 0.342 | 0.162 | 4.45 | -2.343 |
| **With one comorbidity (n=634)** | |  |  |  |  |  |  |  |  |  |  |  |  |  |  |  |  |  |  |
| non-BPHS | 26.7 | 1 (Reference) |  |  |  |  |  | 1 (Reference) |  |  |  |  |  | 1 (Reference) |  |  |  |  |  |
| BPHS | *27.6* | 1.117 (0.751-1.662) | 0.047 | 0.111 | 0.203 | 0.301 | -2.414 | 1.123 (0.754-1.672) | 0.045 | 0.13 | 0.218 | 0.357 | -2.558 | 0.951 (0.629-1.437) | 0.069 | -0.037 | 0.227 | 0.027 | -2.68 |
| **With 2 comorbidities (n=345)** | |  |  |  |  |  |  |  |  |  |  |  |  |  |  |  |  |  |  |
| non-BPHS | 16.4 | 1 (Reference) |  |  |  |  |  | 1 (Reference) |  |  |  |  |  | 1 (Reference) |  |  |  |  |  |
| BPHS | 35.7 | 3.186 (1.735-5.851) *** | 0.075 | 1.065 | 0.293 | 13.188 | -1.954 | 3.222 (1.748-5.936) *** | 0.077 | 1.053 | 0.305 | 11.927 | -1.927 | 2.414 (1.276-4.570) *** | 0.146 | 0.795 | 0.32 | 6.185 | -2.668 |
| **With ≥ 3 comorbidities (n=97)** | |  |  |  |  |  |  |  |  |  |  |  |  |  |  |  |  |  |  |
| non-BPHS | 8.7 | 1 (Reference) |  |  |  |  |  | 1 (Reference) |  |  |  |  |  | 1 (Reference) |  |  |  |  |  |
| BPHS | 35.1 | 5.500 (1.174-25.756) * | 0.211 | 1.656 | 0.842 | 3.873 | -2.402 | 5.136 (1.047-25.203) * | 0.236 | 1.684 | 0.844 | 3.977 | -2.579 | 5.500 (1.174-25.756) * | 0.149 | 1.705 | 0.788 | 4.682 | -2.513 |

1 indicates that the non-BPHS group is taken as the reference group in the logistic regression model.

For the variables included in the models, the variance inflation factor (VIF) is less than 5, and there is no evidence of collinearity between the independent variables.

*Indicated p<0.05

**Indicated p<0.01

*** Indicated p<0.001

**Table S4. Model tests, selection of Figure 2 in the manuscript (Details of models from Figure 2)**

|  |  | Model 1 |  |  | Model 2 |  |  | Model 3 |  |
| --- | --- | --- | --- | --- | --- | --- | --- | --- | --- |
|  | **-2 log likelihood，**  **-2LL** | **Hosmer-Lemeshow tests, p** | **Omnibus Tests of Model Coefficients, p** | **-2 log likelihood，**  **-2LL** | **Hosmer-Lemeshow tests, p** | **Omnibus Tests of Model Coefficients, p** | **-2 log likelihood，**  **-2LL** | **Hosmer-Lemeshow tests, p** | **Omnibus Tests of Model Coefficients, p** |
| **Without comorbidity** | 546.9 | - | <0.001 | 521.6 | 0.71 | <0.001 | 509.6 | 0.33 | <0.001 |
| **With comorbidity** | 1270.1 | - | 0.003 | 1235.9 | 0.41 | <0.001 | 1204.4 | 0.91 | <0.001 |
| **With one comorbidity** | 743.1 | - | 0.807 | 665.5 | 0.87 | 0.614 | 658.2 | 0.92 | 0.188 |
| **With 2 comorbidities** | 404.2 | - | <0.001 | 397.8 | 0.31 | 0.032 | 386.6 | 0.98 | 0.001 |
| **With ≥ 3 comorbidities** | 109.5 | - | 0.008 | 98.5 | 0.90 | 0.041 | 95.4 | 0.71 | 0.032 |

The Hosmer-Lemesho goodness-of-fit test (H-L test) can be used to evaluate whether the model makes full use of the available information to maximize the fit of the model, explaining the variance of the model.

This study can indicate that the model fit superiority effect is good if P>0.05, and can model construction is poor if P<0.05.

The -2 log likelihood (-2LL) is an important index for model evaluation, the smaller the value, the better, which can be used for different models to evaluate the effect.

Model 1 did not adjust for any confounding variables, so p-values for the Hosmer-Lemeshow tests were not available.
